# Pulmonary artery sarcoma: an unexpected settler in the right ventricular outflow tract

**DOI:** 10.1186/s13019-023-02274-6

**Published:** 2023-05-11

**Authors:** Hui-min Hu, Yi-dan Li, Chang-wei Wei, Yan liu, Xiu-zhang lv, Yuan-hua Yang

**Affiliations:** 1 Department of Ultrasound, Beijing Chao-Yang Hospital, Capital Medical University, No. 8 Gongti South Road, Chaoyang District, Beijing, 100020 China; 2 Department of Anesthesia, Beijing Chao-Yang Hospital, Capital Medical University, Beijing, 100020 China; 3 Department of Cardiac Surgery, Heart Center, Beijing Chao-Yang Hospital, Capital Medical University, Beijing, 100020 China; 4 Department of Respiratory and Critical Care Medicine, Beijing Chao-Yang Hospital, Capital Medical University, Beijing, 100020 China

**Keywords:** Pulmonary artery sarcoma, Echocardiography, Right ventricular outflow tract, Case report

## Abstract

Pulmonary artery sarcoma (PAS) is a sporadic malignant tumor that mainly originates from the pulmonary arteries. However, PAS may also involve the right ventricular outflow tract (RVOT) and lead to obstruction, syncope, or sudden death. Early diagnosis and complete surgical resection are essential to prolong survival and improve the quality of life of patients with PAS. Herein, we report a case of a young female patient admitted for pulmonary malignancy and acute pulmonary embolism. The patient had a mass in the RVOT, which was detected by transthoracic echocardiography. Computed tomography and magnetic resonance imaging revealed the invasion depth and extent of the lesions. Surgical resection improved hemodynamics, while pathological and immunohistochemical tests confirmed the diagnosis of a pulmonary artery sarcoma. Local recurrence was detected in the adjacent tissues about two months after the surgery. Given the potential risk of reoperation, the patient was suggested to undergo conservative treatment.

**Supplementary Information:**

The online version contains supplementary material available at 10.1186/s13019-023-02274-6.

## Introduction

Pulmonary artery sarcoma (PAS) is a rare and lethal malignancy that primarily originates from the main pulmonary arteries or pulmonary valve, but may also involve the right ventricular outflow tract (RVOT) [1, 2]. Furthermore, the anatomical location of cardiac sarcomas determines the clinical presentation, treatment, and prognosis of the disease [3]. Masses involving the RVOT may result in obstruction and sudden death. Therefore, early diagnosis is essential for the long-term prognosis of the disease, contributing to a better quality of life. Due to the similar symptoms and imaging findings between PAS and pulmonary thromboembolism (PTE), and since both conditions can develop in some cases simultaneously, the diagnosis and treatment of PAS may often be missed or delayed. Furthermore, it is essential to distinguish PAS from primary cardiac tumors when the RVOT is involved and combine various imaging methods to facilitate differential diagnosis and exact tumor localization.

## Case report

A 34-year-old female presented with a chief complaint of exertional chest tightness for two weeks and chronic cough for more than one year. Computed tomography (CT) performed at an outside hospital led to the diagnosis of acute pulmonary embolism. After anticoagulation treatment for four days, the patient was admitted to the respiratory department. Physical examination upon admission revealed no positive signs. However, a progressively enlarging lesion in the middle lobe of the right lung had been initially detected via CT one year before. More specifically, positron emission tomography-CT (PET-CT) images had shown increased tracer uptake in the lesion with multiple hypermetabolic foci in the right lung, suspected to be metastasis. After transbronchial lung biopsy (TBLB), histologic examination demonstrated minimal spindle cell with typical atypia on inflammatory background. Immunohistochemical techniques revealed that the proliferating spindle cells were positive for Vimentin, P53, and Ki-67 but negative for CK. Consequently, the patient was diagnosed with inflammatory myofibroblastic tumor (IMT) with multiple metastases in the right lung. Following treatment with anlotinib for approximately half a year, symptoms were relieved and gradually recovered, and the largest mass in the computed tomography (CT) images was reduced significantly. However, the patient discontinued the medication just one month prior to admission, and symptoms of chest tightness and shortness of breath reappeared approximately two weeks after. Pulmonary CT re-examination at an outside hospital indicated that the distal branch of the right upper pulmonary artery was poorly visualized, and the surrounding soft tissue, which initially appeared as an opaque shadow, was considered a metastasis. Nodular filling defects were detected in both the pulmonary trunk and left lower pulmonary artery. A small amount of fluid was also observed in the right thoracic cavity and pericardium.

**Fig. 1:**
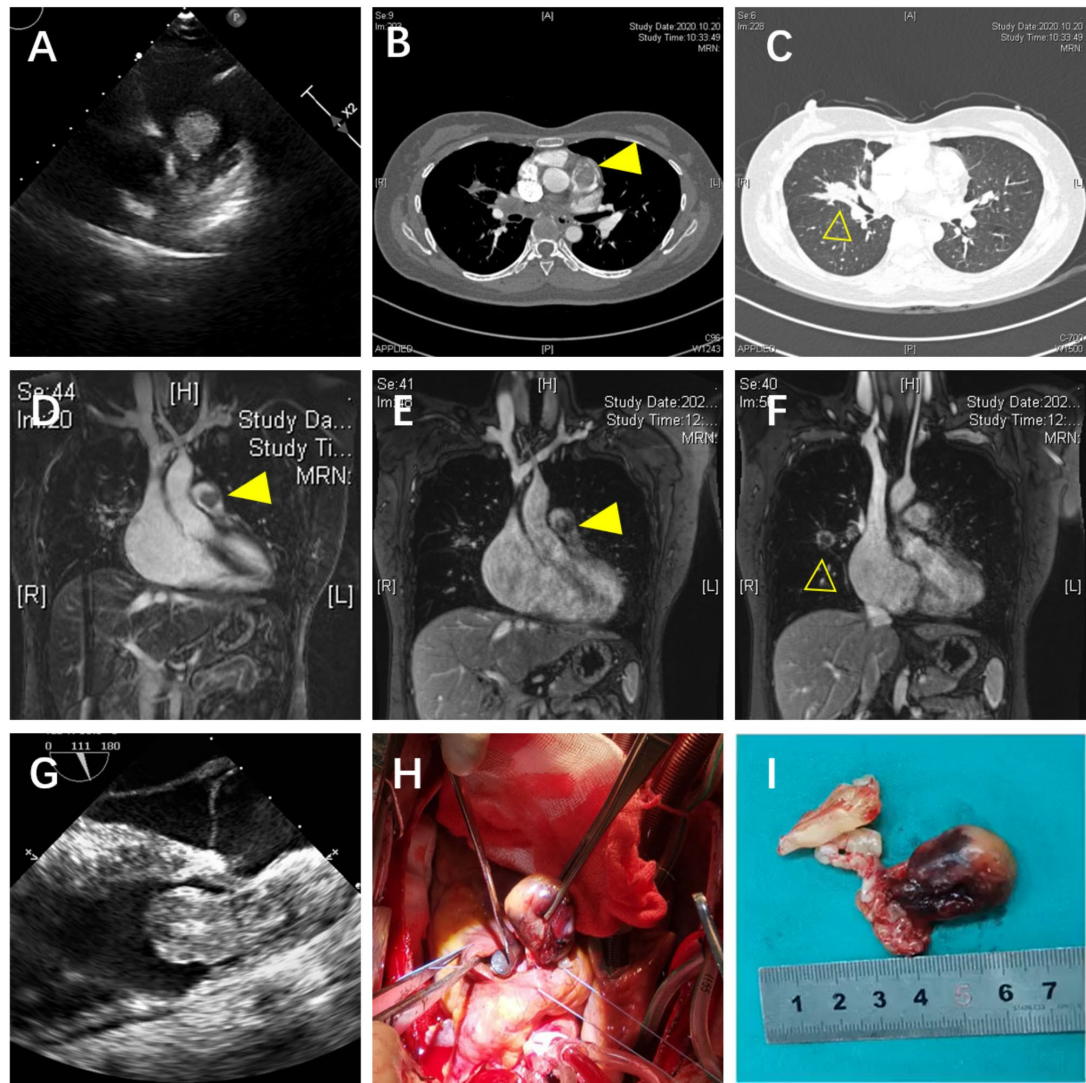
Transthoracic echocardiography (TTE) showing the space-occupying lesions of the right ventricular outflow tract (RVOT) (A). Computed tomography pulmonary angiogram (CTPA) revealed that RVOT obstruction was caused by the mass (B) and the presence of lesions in the right lung (C). The tumor was poorly demarcated with the right ventricular wall and enhanced on MR images of the pulmonary artery (D-E). Inhomogeneous enhancement nodules in the middle lobe of the right lung (F). Transesophageal echocardiography (TEE) showing the multiple pedicled masses with hyperechoic capsule (G). Intraoperative and gross appearance of the tumors (H, I)

Laboratory results on admission showed abnormal liver function (ALT: 54 U/L, AST: 79 U/L, GTT: 104 U/L) and an elevated plasma level of the N-terminal pro-brain natriuretic peptide (1155 pg/ml). Routine laboratory examinations, including coagulation and fibrinolysis index profile, were normal. Continuous anticoagulation was used together with prophylactic liver protecting drugs. Two days after admission, the echocardiogram revealed a pedunculated mass in the RVOT, which was closely related to the pulmonary valve, and protruded to the pulmonary artery during systole (Fig. 1 [A], Movie I in the Additional files). Consequently, the RVOT obstruction was evaluated by a tricuspid regurgitation gradient of 62 mmHg, a peak transpulmonary valve gradient of 39.7 mmHg, right ventricular dysfunction (TAPSE: 14 mm), right heart dilatation (58*66 mm), and right ventricular hypertrophy (5.2 mm), as well as trivial pericardial effusion. Computed tomography pulmonary angiogram (CTPA) performed at the same day demonstrated acute pulmonary embolism in the left lower pulmonary artery branch. Thrombosis or tumor thrombosis was suspected in the main pulmonary artery involving the RVOT (Fig. 1 [B]). It was also apparent that multiple abnormal shadows in the middle lobe of the right lung and lymph nodes in the right hilum and posterior mediastinum were metastatic lesions (Fig. 1 [C]). After reviewing the patient’s previous data together with CTPA from the outside hospital, PET/CT, and enhanced chest CT, we identified that the mass had already been present for more than one year and showed progressive enlargement with no response to anlotinib. We inferred that PAS was missed either due to its small size or because the therapeutic focus was predominantly on the pre-existing lung lesions.

Nonetheless, the above findings encouraged the physician to perform additional investigations to the pulmonary artery using magnetic resonance imaging (MRI) to further evaluate its relationship with the pulmonary valve and right ventricular wall and optimize tumor resection. CMR demonstrated that the tumor was located around the pulmonary valve and extended to the RVOT. The tumor showed iso-signal intensity on T1-weighted and T2-weighted images and poor demarcation with the right ventricular wall. The majority of the lesion and regional wall showed sustained enhancement during the delayed phase (Fig. 1 [D-E]). MRI also presented T2 isointense nodules in the right hilar area and the middle lobe of the right lung and mild inhomogeneous enhancement (Fig. 1 [F]). Multiple enlarged lymph nodes in the mediastinum were fused in clumps with continuous inhomogeneous enhancement. Computed tomography venography of bilateral lower extremities excluded deep vein thrombosis (DVT).

In an attempt to relieve right ventricular outflow obstruction, cardiac surgery was performed to resect the right ventricular mass under intraoperative transesophageal echocardiogram monitoring and evaluation (Fig. 1 [G], Movie II in the Additional files). Three well-capsulated masses (approximately 3.5*2 cm, 1.5*1.0 cm, and 0.5*0.5 cm, respectively) adhered to the bottom of the interventricular septum in the right ventricular via pedicles (Fig. 1 [H, I]). Pathological examinations revealed that the tumors consisted of spindle-shaped cells. Immunohistochemical stains showed positivity for FLI-1, SMA (partial+), CD34 (partial+), CD31 (vascular+), KI67 (40% +), and Vimentin (+). In contrast, ERG, FVIII, S-100, SOX10, CK7, CK20, EMA, and desmin were negative. Based on the above findings, the primary diagnosis was pulmonary artery sarcoma.

**Fig. 2:**
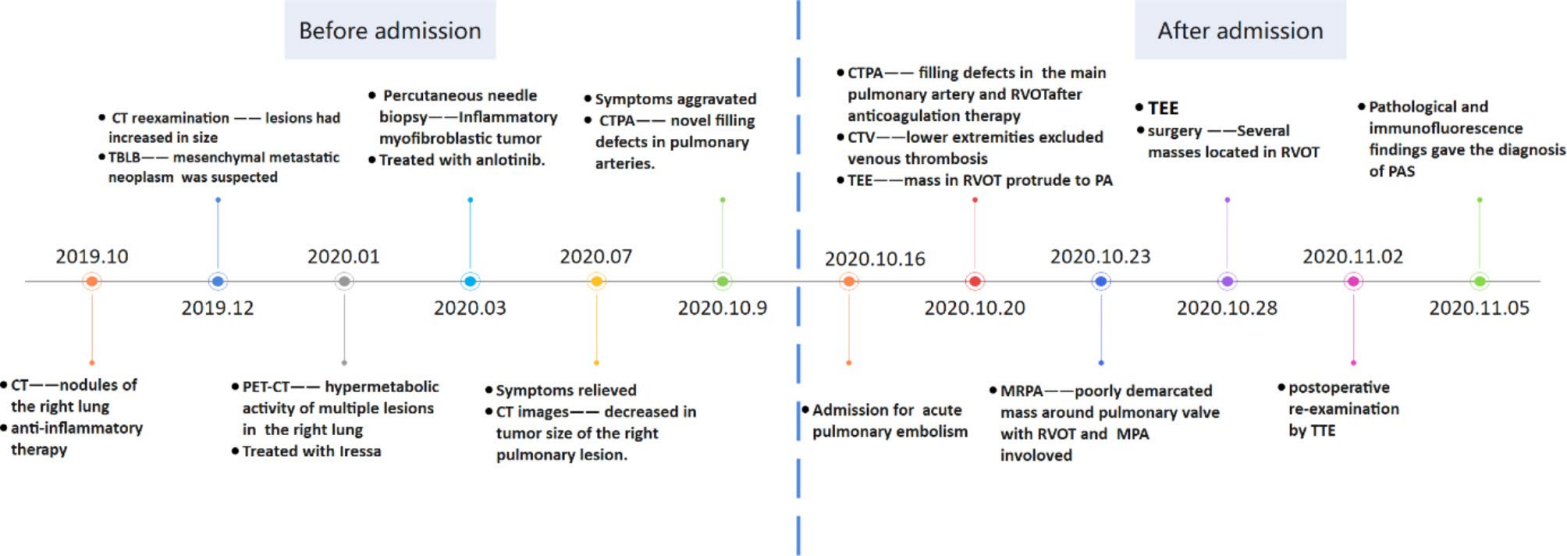
Timeline of significant events before and after admission

After surgery, the right ventricular function (TAPSE: 15.2 mm) improved gradually following relief of the obstruction. Furthermore, echocardiography revealed that the tricuspid regurgitation gradient (49 mmHg) and peak transpulmonary gradient (21.3 mmHg) were significantly reduced. However, seven weeks after discharge, the patient presented to the emergency center again with a complaint of chest tightness and hemoptysis. Consequently, conservative management was advised because tumor recurrence was detected around the primary site, and poor prognosis was expected due to the recurrent and infiltrative lesions (Fig. 2).

## Discussion

PAS is a malignant mesenchymal tumor that originates from the pulmonary trunk and pulmonary arteries intima and may partially involve the pulmonary valve and right ventricular outflow tract. Its prevalence is approximately 0.001–0.030%, and prognosis is poor if left untreated [4]. Clinical presentation and imaging findings of pulmonary artery sarcomas are similar to those caused by pulmonary thrombosis (PE), leading to a high misdiagnosis rate [5]. Indeed, PAS is usually obscured when acute or chronic pulmonary thromboembolism with a tendency for thrombosis is also present [6–8]. A similar case reported by Xu et al. showed the retrograde extension of sarcomas to the pulmonary valve and right ventricle, which were initially misdiagnosed as PTE by CTPA and eventually corrected by echocardiography [9]. Yeung et al. outlined the differences between PAS and PE on echocardiograms, including mobility, absence of echo lucent areas, bulging rather than linear morphology, and attachment to the pulmonary valve or pulmonary artery wall [10]. According to the mobility of the masses and pedicles attached to the interventricular septum and echocardiogram findings, the masses located in the RVOT were diagnosed as tumors instead of thrombosis, which was consistent with the findings of Wang et al. [11]. Thus, echocardiography is a valuable imaging modality for the differential diagnosis of PAS and PTE involved in the pulmonary trunk and RVOT.

In our case, the growth pattern of the tumor originated from the RVOT to the pulmonary valve; hence, it was essential to differentiate this tumor from cardiac tumors such as cardiac myxoma and cardiac sarcoma. The majority of cardiac angiosarcomas occur in the right atrial and are primarily non-mobile with a broad-based attachment to the endocardial surface [12]. Al Umairi et al. reported a case of cardiac hemangioma diagnosed by CMR before surgery and demonstrated that CMR might contribute to the differential diagnosis of cardiac tumors composed of different components, such as fibromas, rhabdomyomas, lipomas, and myxomas [13]. In the present case, ultrasonographic findings on the mobility and pedicles of the masses were similar to those of cardiac myxoma, and thus their nature could not be elucidated. Lung tumors with a similar histological pattern are predisposed to be multiple lesions from the same tissue of origin. When masses develop in the RVOT or pulmonary artery with numerous concurrent lesions in the lung, particular vigilance is required to confirm the possibility of a tumor. The probability of death was increased by 46% with every doubling of the time from onset to diagnosis [4]. In this case, it is a pity that the tumor in the RVOT remained unnoticed and untreated, thus leading to the expected adverse outcomes presented above. Despite the fact that their efficacy is not yet conclusive, surgical treatment offers the best opportunity for symptom relief and improved long-term outcomes [14]. Thereby early detection is necessary to improve survival rates.

In the present study, lesions in the right lung were detected and diagnosed as IMT, while lesions in the RVOT were considered PAS. Histologically, the background of the former is dominated by plasma cells and lymphocytes, while that of the latter is dominated by spindle cells. In addition, it was previously reported that the main manifestations of IMT involving the valve are valve thickening and inflammatory cell infiltration [15], which is different from the pedicled neoplastic lesions reported in the present case. Therefore, despite their immunohistochemistry, which indicates mesenchymal tumors, these lesions can be differentiated based on their cellular components and imaging findings, allowing us to consider them as different types of masses.

## Conclusions

Although cardiac sarcomas and pulmonary artery sarcomas are burdensome malignancies, it is still unnecessary to distinguish between these two types of tumors because of their similar presentation and treatment options. However, a differential diagnosis among pulmonary artery sarcomas, pulmonary thromboembolism, and cardiac myxomas is of utmost significance. Physicians and radiologists should be alert to the possibility of concurrent lesions in the main pulmonary artery or RVOT when peripheral malignant mesenchymal tumors are observed. It is necessary to discriminate lethal lesions and perform timely intervention. Echocardiography can be very helpful for the early detection of tumors in the RVOT and main pulmonary artery. Consequently, consideration of imaging findings and clinicopathological results can facilitate the differential diagnosis of pulmonary thromboembolism and cardiac tumors.

## Electronic supplementary material

Below is the link to the electronic supplementary material.

Supplementary files Movie I: Transthoracic echocardiogram showed activity of the RVOT mass; Movie II: Transesophageal echocardiography showed multiple masses in the RVOT

Additional File 2:

## Data Availability

All data and materials in the case are available per request from the corresponding author on reasonable request.
